# Immobilizing photogenerated electrons from graphitic carbon nitride for an improved visible-light photocatalytic activity

**DOI:** 10.1038/srep22808

**Published:** 2016-03-07

**Authors:** Han Sun, Yue Cao, Leiyu Feng, Yinguang Chen

**Affiliations:** 1 State Key Laboratory of Pollution Control and Resources Reuse, School of Environmental Science and Engineering, Tongji University, Shanghai 200092, People’s Republic of China

## Abstract

Reducing the recombination probability of photogenerated electrons and holes is pivotal in enhancing the photocatalytic ability of graphitic carbon nitride (g-C_3_N_4_). Speeding the departure of photogenerated electrons is the most commonly used method of achieving this. To the best of our knowledge, there is no report on suppressing the recombination of photogenerated electron–hole pairs by immobilizing the electrons with ester functional groups. Here, for the first time the mesoporous g-C_3_N_4_ (mpg-C_3_N_4_) was integrated with polymethyl methacrylate, a polymer abundant in ester groups, which showed a photocatalytic activity unexpectedly higher than that of the original mpg-C_3_N_4_ under visible-light irradiation. Experimental observations, along with theoretical calculations, clarified that the impressive photocatalytic ability of the as-modified mpg-C_3_N_4_ was mainly derived from the immobilization of photogenerated electrons *via* an electron-gripping effect imposed by the ester groups in the polymethyl methacrylate. This novel strategy might also be applied in improving the photocatalytic performance of other semiconductors.

Recently, graphitic carbon nitride (g-C_3_N_4_), a metal-free semiconductor, has received much attention and is rapidly becoming a rising star as a catalyst in many fields^1^,^2^,^3^,^4^. g-C_3_N_4_ is composed of carbon and nitrogen, and features a unique framework of tri-s-triazine linked by tertiary amines, which makes it a promising photocatalyst with a medium band gap, superior chemical and thermal stability and appealing photoelectric properties^3^. Despite these advantages, the photocatalytic activity of g-C_3_N_4_ is still not competitive, basically owing to the low separation efficiency of photogenerated electron–hole pairs and limited use of visible light^5^,^6^. Therefore, modifying g-C_3_N_4_ could enhance its visible-light photocatalytic activity and provide access to its inherent advantages.

Until now, numerous protocols have been adopted to improve the photocatalytic performance of g-C_3_N_4_ under visible-light illumination, and one of the core methods lies in reducing the recombination probability of the photogenerated electrons and holes by separating them more efficiently. Accordingly, accelerating the departure of photogenerated electrons is the most commonly used approach^7^,^8^,^9^,^10^,^11^,^12^. In contrast, if the recombination of photogenerated electrons and holes could be suppressed by immobilizing the electrons, rather than driving them away, the photocatalytic ability of g-C_3_N_4_ under visible-light irradiation would also be improved. This proposed strategy has not been documented in the literature, to the best of our knowledge. The immobilization of photogenerated electrons from g-C_3_N_4_, could be accomplished *via* an electron-gripping effect. The ester groups in chemical compounds possess the ability to grip electrons^13^. When the photogenerated electrons are immobilized by ester groups, the photocatalytic performance of g-C_3_N_4_ should be improved.

Polymethyl methacrylate (PMMA) has been widely used in many fields due to its outstanding chemical stability, transparency and biocompatibility^14^. The carbon skeleton of PMMA consists of a saturated C–C backbone with dangling ester groups (Fig. S1)^15^. Nevertheless, because of the abundant ester groups and their electron-gripping effects, PMMA can be easily chemically modified and establish a good affinity to some polymers, making it a superior polymer substrate^15^,^16^,^17^,^18^. Until now, to the best of our knowledge, there has been no report on improving the separation efficiency of photogenerated electron–hole pairs from g-C_3_N_4_ semiconductor under visible light *via* the immobilization of photogenerated electrons by the ester groups in PMMA.

In this work, mesoporous g-C_3_N_4_ (mpg-C_3_N_4_, denoted as MCN for simplicity) was modified by PMMA enriched with ester groups to achieve better photocatalytic ability under the irradiation of visible light. The resulting MCN/PMMA composite (denoted as PMCN) exhibited much better photocatalytic performance during the degradation of an organic dye than MCN. Experimental observations along with theoretical calculations revealed that this enhancement in the photocatalytic ability was mainly because of the immobilization of photogenerated electrons by ester groups which resulted in a lower recombination probability of photogenerated electron–hole pairs, and a better use of visible light caused by a narrower band gap.

## Results

### Morphological and structural characterizations of the photocatalyst

**Figure 1:**
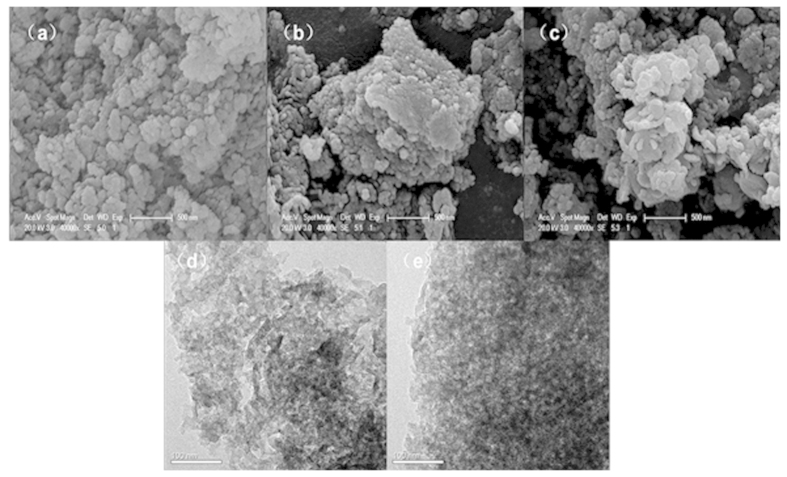
SEM images of (**a**) MCN, and (**b**,**c**) PMCN and HRTEM images of (**d**) MCN and (**e**) PMCN.

In this work, MCN was modified by PMMA *via* a sonochemical process, and the morphological analysis of these as-prepared photocatalysts was conducted by scanning electron microscopy (SEM) and transmission electron microscopy (TEM), as shown in Fig. 1. Figure 1a demonstrates the typical mesoporous structure of MCN, whose surface was composed of tiny spherical and ellipsoidal particles from the use of a silicon template during the synthesis of MCN^19^. When PMMA was introduced onto the MCN, significant changes in the MCN were detected although PMMA accounted for only a small amount of the material in PMCN. PMMA was covered over the surface of MCN and many of the smallest particles MCN tended to conglomerate (Fig. 1b). This was because PMMA is a long chain polymer, and some of MCN particles were interconnected so they appeared to be short bars, with PMMA functioning as a polymer linkage between the carbon nitride particles (Fig. 1c). The interaction between MCN and PMMA can be further verified by the TEM images (Fig. 1(d,e)). It can be clearly observed that MCN appeared to be much broken pieces while PMCN was in a more complete form with relatively smooth edge due to the gluing of PMMA distributed in the composite, indicative of a good interaction between MCN and PMMA.

**Figure 2:**
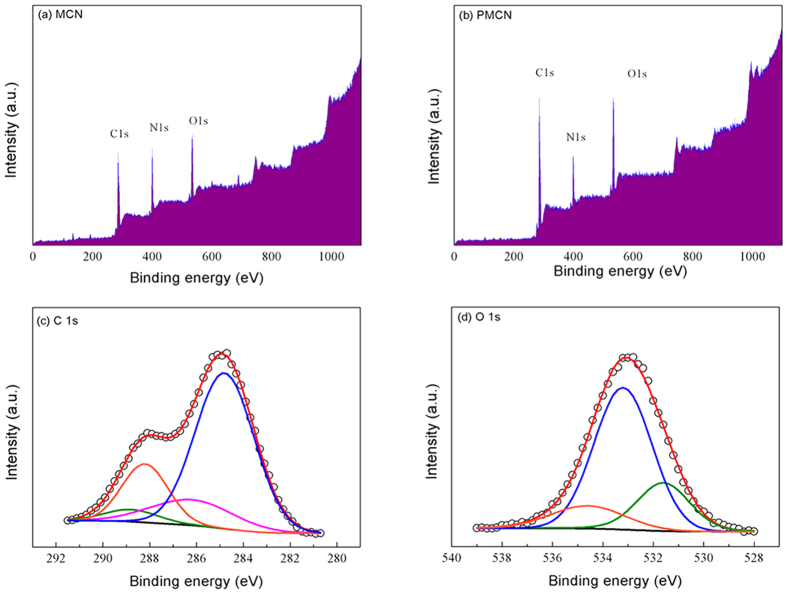
XPS spectra for (**a**) MCN and (**b**) PMCN, high resolution (**c**) C 1 s and (**d**) O 1 s spectra of PMCN fitted with multiplet peaks (the empty circle lines represent the raw data and the red solid lines are best fitted to the raw data).

The chemical states of the component elements on the surface of current photocatalysts were evident from the X-ray photoelectron spectroscopic (XPS) measurements. The survey spectrum of MCN, displayed in Fig. 2a, shows that the semiconductor was composed of carbon, nitrogen and a limited amount of oxygen inevitably absorbed on the surface, as evidenced by the binding energy peaks at 284.5, 399.5 and 532.5 eV, respectively. When PMMA was implanted into MCN, forming the PMCN composite, the amount of carbon and oxygen became noticeably higher than those of the original carbon nitride (Fig. 2b), because PMMA consists only of C, O and H. Figure 2c presents the high resolution C 1 s spectrum of PMCN, and could be fitted using four peaks. The peak located at 288.2 eV was identified as an sp2-bonded carbon (N–C=N), the fundamental building block of the carbon nitride^20^, while the peak at 288.8 eV has been assigned to the carbon from the ester groups (O–C=O) of PMMA. The other C–O species in PMCN gave another binding energy peak at 286.2 eV^21^. The peak centred at 284.8 eV could be assigned to C–C bonded carbons, which was attibuted to the graphitic carbon of MCN and PMMA. The deconvolution of the O 1 s spectrum, shown in Fig. 2d, resulted in three peaks which represented three distinguishable types of oxygen in the composite. The peak at 531.6 eV belonged to the C=O from the ester groups and the peak at 533.2 eV was derived from the C–O bonded carbons^22^, both of which come from the PMMA, demonstrating that both MCN and PMMA are present in the final product. Another small peak in the spectrum at 534.6 eV was identified as chemisorbed oxygen.

The X-ray diffraction (XRD) patterns of MCN and PMCN are illustrated in Fig. S2. Two strong peaks were observed in the XRD profiles of both samples at about 13.1° and 27.3°, which correspond to the (100) and (002) planes, respectively. The diffraction peak at 13.1° indicated a typical in-plane structural packing motif, whereas that at 27.3° was attributed to the interlayer stacking of aromatic systems^23^. When the XRD patterns of MCN and its composite were compared, no new peaks were identified; implying that there were no marked changes in the crystal structure of MCN, and the introduction of PMMA had little influence on its interlayer and in-plane structures.

**Figure 3:**
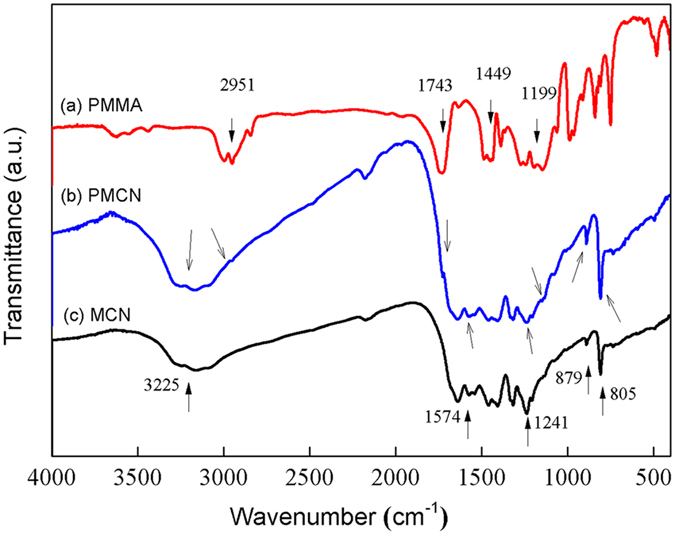
FTIR spectra for (**a**) PMMA, (**b**) PMCN and (**c**) MCN.

The molecular structure of PMCN was verified by Fourier Transform Infrared Spectroscopy (FTIR), and is depicted in Fig. 3. The FTIR characterization of PMMA and MCN has been included for reference. Two peaks owing to the presence of PMMA were observed at 1743 and 1199 cm^−1^, which have been assigned to stretching modes of the C=O and –O–C– of the ester groups. The bands at 2951 and 1449 cm^−1^ were respectively assigned to the –CH– and –CH_3_ stretching modes^24^,^25^. For the MCN, the modes typical of CN heterocycles were detected and characteristic peaks appeared at 1241 and 1574 cm^−1^. In addition, the existence of triazine structure of carbon nitride could be confirmed by the bands at 805 and 879 cm^−1^. As for the FTIR spectrum of PMCN, although the characteristic bands assigned to C=O, –O–C– and –CH– still appeared, their intensities were comparatively weak as PMMA was only a minor constituent. The hydrogen bond adsorption peak at around 3225 cm^−1^ was enhanced, revealing a strong interaction between MCN and PMMA which can also be demonstrated by the absence of peak at 1743 cm^−1^ in the FTIR spectrum of physical mixture of MCN and PMMA (Fig. S3). Carbon nitride is an electron-rich polymeric semiconductor because of the lone-pair electrons from the nitrogen atoms, leading to an electronegative surface^26^. Meanwhile, PMMA is abundant in ester groups, which have the ability to grip electrons^13^. The macromolecular PMMA has an affinity for the electronegative carbon nitride and is likely to impose an influence on the energy band structure of carbon nitride, which is in turn closely associated with the photocatalytic performance^27^.

### Photocatalytic capability evaluation of PMCN

**Figure 4:**
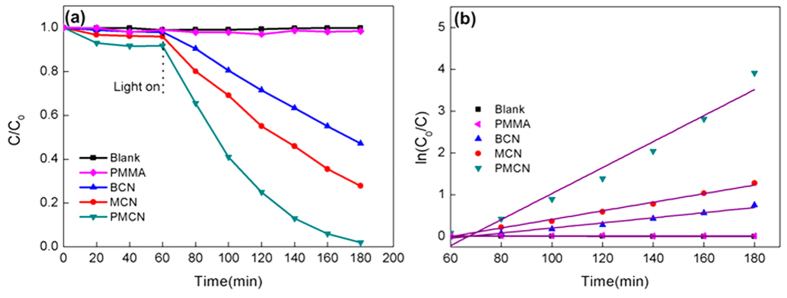
(**a**) Photocatalytic activities and (**b**) reaction constants of resultant samples under visible light during the decolorization process and its inset is the first-order plots for RhB decolorization.

The photocatalytic performance of MCN and PMCN were tested in a series of experiments based on rhodamine B (RhB) degradation under visible light. As a control, the photocatalytic ability of bulk g-C_3_N_4_ (denoted as BCN) was also evaluated. As shown in Fig. 4, the self-decomposition of RhB (blank sample) and the photodegradation ability of PMMA toward RhB can be ignored due to the negligible amount of degradation over the entire irradiation time. Compared with BCN, MCN showed improved degradation of RhB, possibly because the mesoporous carbon nitride has a larger specific surface area and better optical adsorption ability, which have been verified through a series of characterizations for BCN (Fig. S4) and was in good agreement with previously reported literature^28^. With the introduction of PMMA, the PMCN sample exhibited much better photocatalytic performance than MCN, and more than 98% of RhB was decolorized in the presence of the photocatalyst, verifying that PMMA significantly improved the photocatalytic ability of current available materials. In the current study, the photocatalytic activity of PMCN was also evaluated by the colourless phenol, and found that its photodegradation efficiency under the visible light was much higher than those with MCN and BCN (Fig. S5), revealing that the synthesized PMCN was an excellent visible-light photocatalyst.

To gain quantitative insight into the reaction kinetics of RhB decolorization, a first-order model was applied here to fit the above-explained experimental data and the first-order plots are displayed in the inset of Fig. 4b. The fitted plots of all the tested samples, namely the plot of the irradiation time (*t*) against ln(*C*_*0*_*/C*), have shown a shape of straight line. Accordingly, the reaction constant *k* values which can help evaluate the decolorization rates were concluded in Fig. 4b. It can be found that the as-prepared PMCN exhibited the best performance during the decolorization of RhB and its *k* value was calculated to be 0.0311 min^−1^, which was almost 3.0-fold higher than that of MCN (0.0103 min^−1^) and 5.0-fold higher than that of BCN (0.0061 min^−1^). Therefore, the present synthesized PMCN can be confirmed to have superior performance in the photocatalytic decolorization over RhB.

Apart from the decolourization of organic dye under the irradiation of visible light, the TOC removal efficiency was evaluated to deeper illuminate the degradation capabilities of our photocatalyst (Fig. S6). Among all the tested samples, PMCN presented the most efficient photodegradation of RhB with a TOC removal efficiency of 57.0%, followed by MCN (29.5%) and BCN (24.0%). This experiment also showed that RhB failed to decompose without the photocatalysts, revealing PMCN is able to mineralize RhB during the decolourization process.

The photodegradation stability under the irradiation of visible light is a key property for the photocatalyst of organic pollutants. Therefore, the photocatalytic capability of recycled PMCN was evaluated to determine its photocatalytic stability. As shown in Fig. S7(a), When the PMCN photocatalyst was used three times, its ability on RhB decolourization can maintain a relative high level. Moreover, both XRD and XPS spectra revealed that there was neither structural nor compositional change of PMCN after the cycling tests (Fig. S7(b,c)). Therefore, it can be confirmed that MCN modified with PMMA exhibited good photocatalytic stability under the visible light.

### Understanding the photocatalytic activity of MCN improved by PMMA

**Figure 5:**
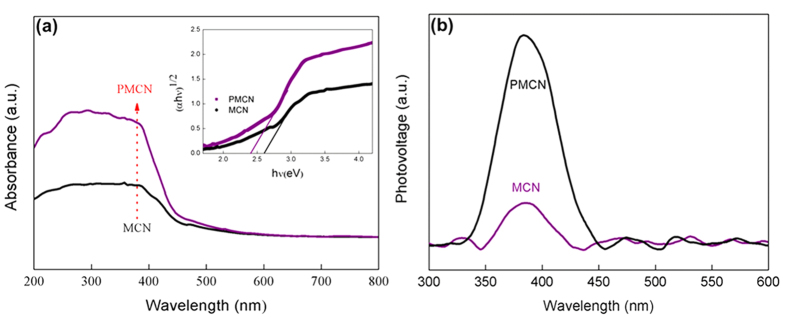
(**a**) Optical absorption of MCN and PMCN. UV-vis DRS of catalytic samples and curve of (Ahv)^1/2^-(hv) for MCN and PMCN (inset) (**b**) Surface photovoltage spectra (SPV) of PMCN composite and MCN.

The photocatalytic activity of semiconductor is highly correlated with its optical absorption and the separation efficiency of the photogenerated electron–hole pairs. In this study, the UV-vis diffuse reflectance spectrum was used to investigate the optical properties of the photocatalysts (Fig. 5). MCN absorbed in both visible and ultraviolet light, and the absorption edge was observed at around 460 nm, which was in good agreement with the widely accepted band gap of around 2.70 eV. In contrast with the pure mpg-C_3_N_4_, the absorption intensity of PMCN was observed to be noticeably enhanced with the loading of PMMA, indicative of a better photocatalytic performance for PMCN because more electron-hole pairs can be generated through the visible light excitation of as-prepared composite during the photocatalytic process. As a result, the PMCN material possessed a better photocatalytic capability under visible light.

PMMA is a transparent optical material with a high transmittance of 92%, with the 8% loss mainly the result of reflection^29^. Thus, unlike other conjugated polymers which have high absorption coefficients in the visible-infrared region^7^, PMMA does not contribute to the visible-light absorption of our composite owing to PMMA’s optical characteristics. However, these results show that PMCN has better performance upon optical absorption than MCN. This means that the introduction of PMMA makes carbon nitride a better photon acceptor under visible-light irradiation, which can be further demonstrated by examining the band gap. The band gap energy is related to the optical absorption in semiconductors. In this study, the band gap of PMCN was calculated by the equation (1):


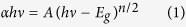


where*α, h, ν, E*_*g*_*, A* and *A* are the absorption coefficient, Plank constant, light frequency, band gap energy and a constant, respectively^30^. In this study, *n* is 4 according to the optical transition type of the constituents, MCN and PMMA^31^,^32^. As shown in the inset of Fig. 5, a plot of *αhν*^1/2^ *– hν* for PMCN revealed a band gap energy of around 2.38 eV, which is lower than MCN (2.70 eV). A narrower band gap (2.38 versus 2.70 eV) endowed the PMCN material will aid the absorption of visible light, and is consistent with the results given by the UV-Vis measurements.

The surface photovoltage spectroscopy (SPV) technique was employed to reveal the kinetic behaviors of the photogenerated charge carriers in the as-prepared PMCN samples. The signal of surface photovoltage (SPV) can be attributed to the variations of surface potential barriers during the light irradiation, which can identify the light-responsive wavelength range and the separation efficiency of the electron–hole pairs in the photocatalysts^33^. From Fig. 5b, it can be found that the SPV signal of MCN was significantly increased with the introduction of PMMA, confirming that more photoexicited electrons and holes were produced and then separated more efficiently in PMCN during the phtocatalytic process. Therefore, PMCN possessed a stronger photocatalytic activity than that of MCN.

**Figure 6:**
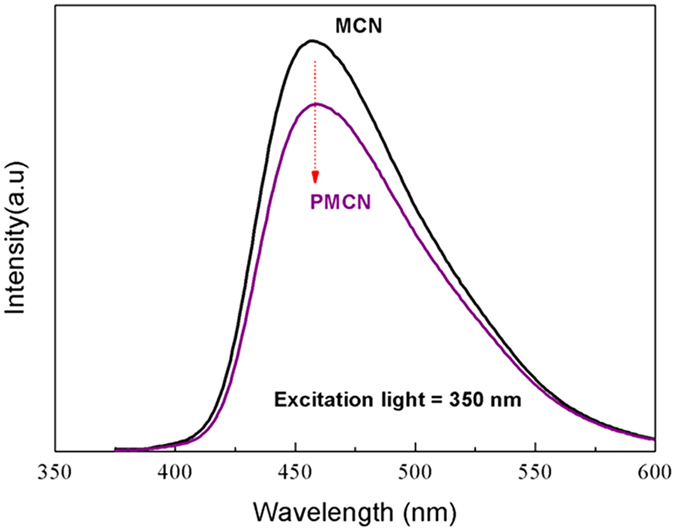
Photoluminescence spectra for current photocatalysts.

To further investigate the photocatalytic properties of our catalysts, the photoluminescence (PL), which characterizes the separation efficiency of photogenerated electron–hole pairs in semiconductors was measured and is shown in Fig. 6. The main emission peak was located at 460 nm, which was attributed to the recombination of photogenerated electron–hole pairs^20^. Further exploration revealed that the PL density for PMCN was much lower than that for MCN, indicting an obvious reduction in the recombination probability between photogenerated electrons and holes. This lower recombination probability of photogenerated electrons and holes from MCN can be attributed to the electron-gripping effect derived from the abundant ester groups of PMMA. The photocatalytic performance of MCN was expected to be improved with the introduction of PMMA owing to this, more efficient separation of photogenerated electrons and holes.

**Figure 7:**
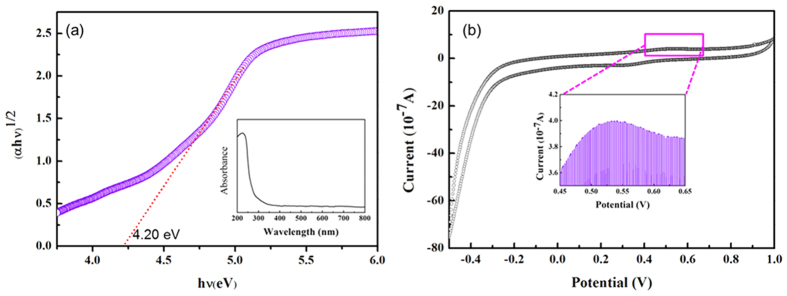
Calculations for band structure of PMMA. (**a**) UV-vis DRS of PMMA and its curve of (Ahv)^1/2^-(hv) and (**b**) cyclic voltammogram of PMMA (0.1 M NaSO_4_ solution and scan rate at 10 mV/s).

**Figure 8:**
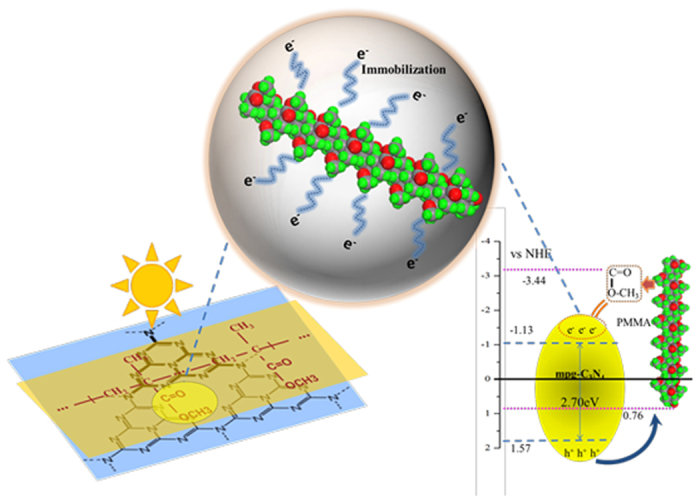
Mechanisms for enhanced photocatalytic activity of MCN by PMMA.

To explore this improvement in the separation of photogenerated electron–hole pairs of the PMCN, the energy band structures of the two constituents in the PMCN photocatalyst were determined, to understand fully the influence of the PMMA on the MCN. MCN has a widely accepted band gap of 2.70 eV with the valence band (VB) and conduction band (CB) at −1.13 eV and +1.57 eV versus the Normal Hydrogen Electrode (NHE), respectively. The HOMO–LUMO energy gap of PMMA and its position, however, have never been reported and have been calculated for the first time in this study. As displayed in Fig. 7a, the HOMO–LUMO gap of the PMMA polymer was found to be ca. 4.20 eV, which was significantly wider than in MCN, meaning that PMMA would have comparatively limited excitation of electrons and holes when irradiated by visible light as a consequence of this high energy threshold. The HOMO and LUMO potentials can be obtained from the oxidation potentials (E_ox_) from cyclic voltammetry (CV)^8^,^34^. Thus, CV experiments of the catalysts were performed to give detailed HOMO–LUMO information, to understand the behaviour of photogenerated electrons and holes during the photocatalytic reaction. Based on the CV plot shown in Fig. 7(b), the HOMO potential of PMMA was +0.53 eV vs. Ag/AgCl electrode, or +0.76 eV vs. NHE. The LUMO potential of PMMA in this work was therefore calculated to be −3.44 eV vs. NHE using the eq. (2) and the band energy structures of both MCN and PMMA are illustrated in Fig. 8.


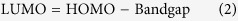


PMMA is a polymer made from methyl acrylate monomers, and its carbon skeleton is composed of a saturated C–C backbone with dangling ester groups. It has been documented that some polymers like polyaniline accelerate the departure of electrons to enhance the efficient separation of photoinduced electron–hole pairs from MCN^7^. In this work, however, based on the highlighted photocatalytic capability of PMCN, it could be confirmed that PMMA has a different mode of enhancing the photocatalytic performance of MCN because PMMA could easily induce a departure of the photogenerated electrons so that the recombination probability of the photoelectrons and holes can be suppressed.

When irradiated with visible light, MCN produces photogenerated electron–hole pairs. Although PMMA makes a negligible contribution to the light absorption of PMCN because of its wide band gap, its addition to the catalyst still improves the photocatalytic performance of MCN in the mineralization of RhB from 29.5% to 57.0%. This enhancement in the photocatalytic ability of MCN comes from a number of ways in which the photogenerated holes and electrons are affected by PMMA during the photocatalytic reaction. First, the ester groups from PMMA gripped the photogenerated electrons from MCN and restricted their movement. This reduced the recombination of electrons and holes in the valance band of MCN. Second, the VB of MCN is 1.57 eV and the HOMO of PMMA is 0.76 eV, and this difference between the band energy positions drives the transfer of photogenerated holes from the VB of MCN to the HOMO of PMMA, which further inhibits their recombination with photogenerated electrons. Consequently, this immobilization of the photogenerated electrons by ester groups and the transfer of photogenerated holes from MCN to PMMA make synergistic contributions to the efficient separation of photogenerated electrons and holes in MCN (as the proposed principle shown in Fig. 8). The photocatalytic activity of MCN was therefore improved by the introduction of PMMA when irradiated with visible light.

**Figure 9:**
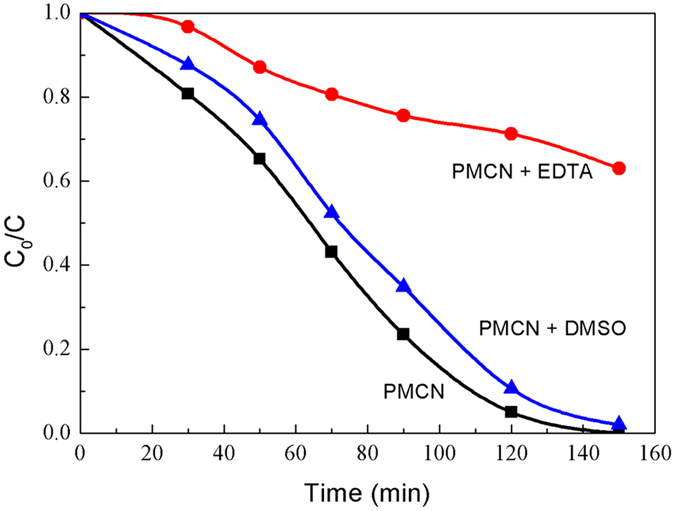
Influence of DMSO and EDTA on RhB photodegradation by PMCN.

As the photogenerated electrons are efficiently immobilized in the MCN, the main active species to degrade RhB under visible light must be the photogenerated holes. To verify whether the photogenerated electrons or holes were the active players in the photodegradation of RhB, the electrons were captured by dimethylsulfoxide (DMSO) and holes by ethylene diamine tetraacetic acid (EDTA) during the photocatalytic process^35^,^36^. As seen in Fig. 9, the photocatalytic performance of PMCN under visible light was scarcely influenced by the addition of electron trapper (DMSO) with a decolourization efficiency of more than 98%, which demonstrated that the photogenerated electrons have been shielded and make little contribution to the degradation of RhB. The presence of EDTA in the current photocatalytic systems, however, brought an obvious reduction in the degradation capability of PMCN, showing that the photogenerated holes were the key factor in the decomposition of RhB.

## Discussion

Our work suggested that the sonochemical coupling of MCN and PMMA gave rise to a novel, simple, highly efficient and metal-free visible-light photocatalyst. During the fabrication of the composite photocatalyst (PMCN), the noticeable changes were detected in the MCN morphology when the PMMA was added. Based on the XRD spectrum of PMCN, however, it can be demonstrated that the crystal structure of MCN remained almost unchanged and PMMA did not affect the interlayer and in-plane structures. In the FTIR spectrum of PMCN, although the characteristic bands assigned to different functional groups appeared weaker than those of PMMA, the enhancement in the hydrogen bond adsorption confirmed a strong interaction between the PMMA and MCN. From the XPS spectrum of PMCN, different types of C and O bands further confirmed both MCN and PMMA were present. The photocatalytic ability of PMCN was evaluated by the degradation of RhB under the irradiation of visible light, and it was found that the photocatalytic activity and stability of MCN for RhB decolourization, and even its mineralization, were significantly improved by PMMA. As far as we know, all the reported carbon nitride catalysts with superior photocatalytic activity and durability have been modified by the exterior metal-based materials or some special polymers^1^,^25^,^37^. This pathway by which MCN modified by PMMA improves the photocatalytic activity is different from the commonly used methods.

We further studied the optical absorption of PMCN, and observed an enhanced absorption intensity owing to the introduction of PMMA, which suggested that more electron–hole pairs from the carbon nitride were being generated under visible light. Usually, the optical absorption of semiconductor has a close relationship with the band gap. In this study, the band gap energy of PMCN was calculated to be about 2.38 eV, lower than that of pure carbon nitride, which meant the composite absorbed visible light better, and resulted in a highlighted photocatalytic activity for the degradation of organic dyes. More importantly, the photocatalytic features of present composite were studied by photoluminescence. The decrease of PL density of PMCN compared with MCN indicated that the recombination probability of photogenerated electrons and holes from semiconductors was reduced. To further explore this more efficient separation of photogenerated electron–hole pairs on PMCN, the energy band gaps of the two constituents of the composite were calculated to be about 2.70 and 4.20 eV, which meant that the photogenerated holes could move from MCN to PMMA, and that the photoinduced electrons were immobilized by electron-gripping effects from the ester groups of PMMA, leading to an improved separation of photogenerated electrons and holes.

In summary, the visible-light photocatalytic ability of carbon nitride was improved by immobilizing its photogenerated electrons with ester groups from PMMA. The PMCN material showed impressive photocatalytic activity and stability under irradiation by visible light. This work not only provided an efficient visible-light photocatalyst, but also offered a new strategy in the design of novel catalysts based on the modulation of functional groups, which could be further applied in the modification of other semiconductors for improved photocatalytic ability and stability under visible light.

## Materials and Methods

### Materials

Cyanamide, ammonium bifluoride (NH_4_HF_2_), ethanol and chloroform were purchased from Sinopharm Chemical Reagent Co. Ltd. (China), and 12 nm SiO_2_ hydrosol from Sigma-Aldrich. All the reagents were reagent grade and were used without further purification. All the solutions were prepared with doubly distilled water.

### MCN preparation

The MCN photocatalyst was prepared according to the method as follows. Typically, 10 g of cyanamide was firstly dispersed in a certain amount of water to form transparent cyanamide solution, followed by dropping 4 g of silica template solution (12 nm SiO_2_ hydrosol, Ludox HS40). The mixed solution was then stirred and heated at 70 °C until most of the water was evaporated. The semitransparent dry mixture was placed into the tube furnace congested with Argon and heated at 550 °C for 4 h. The resulting powder was treated with a 4 M NH_4_HF_2_ for 24 h to remove the silica template, and then washed with purified water and ethanol for three times. Finally, the purified powders (MCN) were dried at 70 °C under vacuum overnight. For the reference, the bulk g-C_3_N_4_, named as BCN, was also synthesized by directly heating cyanamide at 550 °C for 4 h under the argon atmosphere.

### Modification of MCN by PMMA

The modification of MCN by PMMA was conducted *via* a sonochemical approach. The typical procedure was as follows: 30 mg of PMMA was dissolved in chloroform (20 mL) to form the transparent solution. Then, 300 mg of MCN powder was added into the above-mentioned solution. After being stirred for 1 h, the resulting suspensions were further treated with sonochemical method for 12 h. The MCN/PMMA composite was ultimately obtained by filtering the suspensions and then dried at 80 °C under vacuum for another 12 h.

### Characterization of synthesized materials

The morphology of current samples was investigated by field emission SEM on a Hitachi S-4800 and JEOL JEM-2010 high resolution electron microscope. The XRD determination was conducted on a Bruker D8 Advance X-ray diffractometer with Cu K-alpha radiation at a scan rate of 1° 2θ s^−1^. The accelerating voltage and applied current were 40 kV and 40 mA, respectively. FTIR survey was carried out on a Nicolet 5700 spectrometer at room temperature. XPS measurements were performed on a Perkin Elmer PHI 5000C ESCA system with a monochromic Al K-alpha X-ray source. The binding energies were calibrated by referencing the C 1 s peak (284.6 eV) to reduce the sample charge effect. Before each analysis, the samples were dried under vacuum at 80 °C. Spectra obtained over a scan range of 0–1100 eV were recorded and stored using the PHI ACCESS data system, and analyzed with XPSPEAK41 software. UV-Vis spectra was performed on a Shimadzu UV-3600 spectrophotometer using barium sulfate as the reference. The surface photovoltage spectroscopy was conducted on a SPV measurement system, which was constituted of a source of monochromatic light, a lock-in amplifier (SR830-DSP) with a light chopper (SR540), a photovoltaic cell, and a computer. The monochromatic light was provided by a 500-W xenon lamp and a grating monochromator (Omni-λ 500). A low chopping frequency of ~23 Hz was used. Nitrogen adsorption-desorption isotherms were measured with a Quantochrome autosorb IQ instrument at liquid nitrogen temperature. The PL spectra of current photocatalysts were collected at room temperature by a Shimadzu RF-5301PC spectrofluorophotometer with an excitation wavelength of 350 nm using a Xenon lamp as a light source. Cyclic voltammetry measurements were conducted on a computer-controlled electrochemical workstation (Autolab PGSTAT 302 N) with a typical three-electrode cell equipped with gas flow systems. A glassy carbon (GC) electrode (3 mm in diameter, Pine Research Instrumentation) used as working electrode, an Ag/AgCl electrode (3 M KCl-filled) as reference electrode, and a platinum wire as counter electrode. An aqueous solution of 0.1 M sodium sulfate was applied as the supporting electrolyte for the measurements. For the fabrication of the working electrode, 50 mg of as-prepared materials, 1 mL chloroform and 0.1 mL Nafion solution (5%) were mixed together and then sonificated for 10 min to make a slurry, which was coated onto the GC electrode and dried under pure nitrogen stream prior to experiments.

### Evaluation of photocatalytic capability

Two typical organic pollutants, RhB and phenol, were selected as the targets to evaluate the photocatalytic activity of as-prepared mpg-C_3_N_4_/PMMA composite in an aqueous solution under the illumination of visible light. The pure carbon nitride (MCN and BCN) was applied as the reference photocatalyst. 30 mg of resultant photocatalysts were dispersed in 50 mL aqueous solution of organic pollutants (50 mg/L) and stirred for 60 min to reach an adsorption-desorption equilibrium in reaction systems. During the photoreaction under the visible light, the suspension was exposed to a 300 W Xenon lamp with a 400 nm optical filter at ambient pressure and 20 °C. At the given intervals of photoreaction, 1 mL suspension was taken and centrifuged to separate the liquid samples from solid photocatalysts. The concentration of RhB was determined *via* a UV-vis spectrophotometer at a wavelength of 550 nm. In order to further investigate the photocatalytic performance of composites, the efficiency of TOC removal was also measured using a Shimadzu TOC-V analyzer. C_0_ and C were the symbolic representations for the concentration of organic pollutants before and after the photocatalytic experiments, respectively. The as-fabricated material was recycled for three times in order to evaluate its photocatalytic stability. Each time after the experiment was completed, the phtotocatalyst was recollected through centrifugation and washed by ethanol and water for three times, respectively, which was aimed to purify the residual organic matters attached on the photocatalyst.

## Additional Information

**How to cite this article**: Sun, H. *et al*. Immobilizing photogenerated electrons from graphitic carbon nitride for an improved visible-light photocatalytic activity. *Sci. Rep.* **6**, 22808; doi: 10.1038/srep22808 (2016).

## Supplementary Material

Supplementary Information
