# Supplementary material for: Immobilizing photogenerated electrons from graphitic carbon nitride for an improved visible-light photocatalytic activity
Source: Sci Rep. 2016 Mar 7;6:22808. doi: 10.1038/srep22808 (PMC4779999; doi:10.1038/srep22808)
Supplement: Supplementary Information [file srep22808-s1.pdf]

Supplementary Information for

Immobilizing photogenerated electrons from graphitic carbon nitride for an improved visible-light photocatalytic activity

Han Sun, Yue Cao, Leiyu Feng*, Yinguang Chen*

State Key Laboratory of Pollution Control and Resources Reuse, School of Environmental Science and Engineering, Tongji University, Shanghai 200092, People's Republic of China

*Corresponding author

E-mail: leiyufeng@tongji.edu.cn, yinguangchen@yahoo.com

Tel: 86-21-65981263

Fax: 86-21-65986313

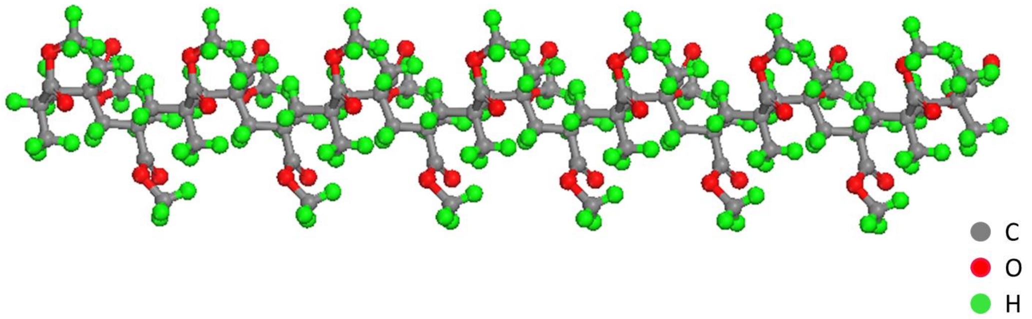

Figure S1. Chemical structure of PMMA

The carbon skeleton of PMMA consists of a saturated C–C backbone with dangling ester groups, which makes it a polymer with inherently inferior conductivity.

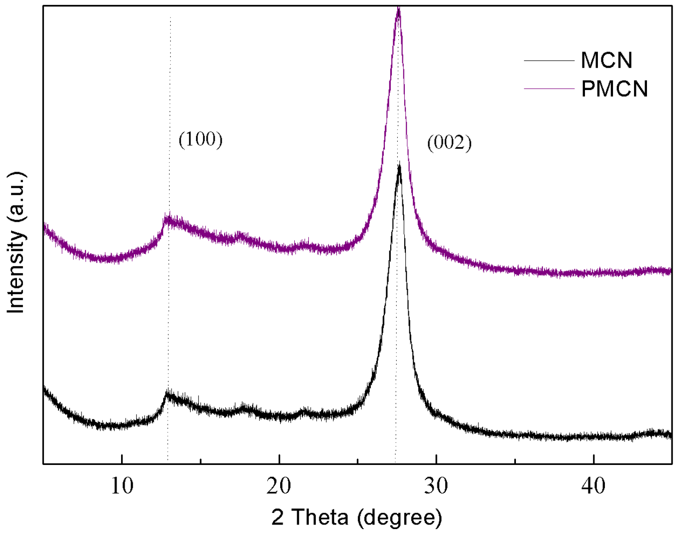

Figure S2. XRD patterns of MCN and PMCN

The X-ray diffraction (XRD) patterns of MCN and PMCN are illustrated in Figure S1. Two strong peaks were observed in the XRD profiles of both samples at about 13.1 ° and 27.3 °, which correspond to the (100) and (002) planes, respectively. The diffraction peak at 13.1 ° indicated a typical in-plane structural packing motif, whereas that at 27.3 ° was attributed to the interlayer stacking of aromatic systems.[1] When the XRD patterns of MCN and its composite were compared, no new peaks were identified; implying that there were no marked changes in the crystal structure of MCN, and the introduction of PMMA had little influence on its interlayer and in-plane structures.

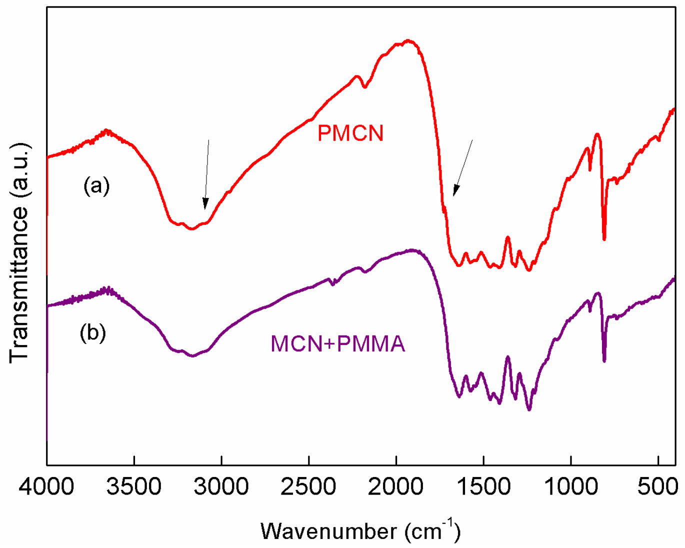

Figure S3. FTIR spectra of (a) PMCN and (b) physical mixture of MCN and PMMA

The FTIR characterization on the physical mixture of MCN and PMMA has been conducted in order to give more information on the interaction between MCN and PMMA. It was found that in the FTIR spectrum of PMCN there were some obvious peaks compared with that from the physical mixture of MCN and PMMA. The hydrogen bond in the as-fabricated PMCN composite is obviously stronger than that in the physical mixture, showing a better interaction between the components in the composite. In addition, in the FTIR spectrum of PMCN a peak assigned to stretching modes of the C=O of the ester groups was observed at 1743 cm^{-1} in the PMCN composite, but not appeared in that of the physical mixture.

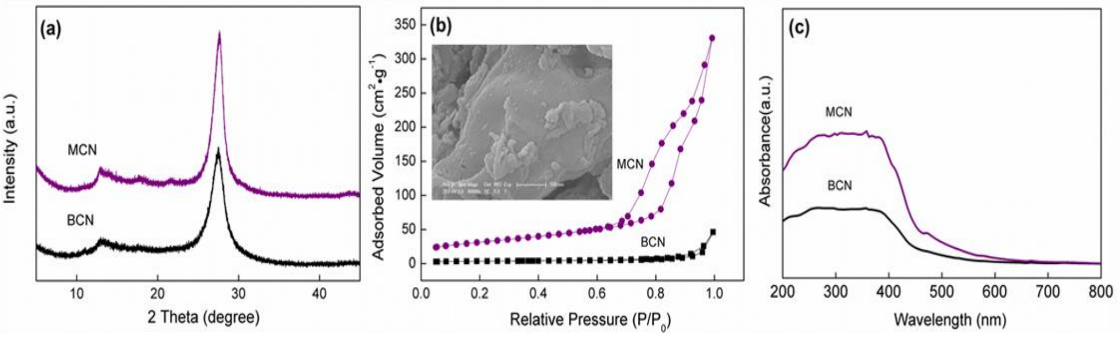

Figure S4. Comparison between BCN and MCN: (a) XRD patterns, (b) N_2 adsorption–desorption isotherms (inset: SEM image of BCN) and (c) UV-Vis spectra

A series of characterizations for BCN were also conducted. Both the specific surface area and optical adsorption ability of MCN were increased compared with those of BCN, leading to a better photocatalytic ability under the visible light.

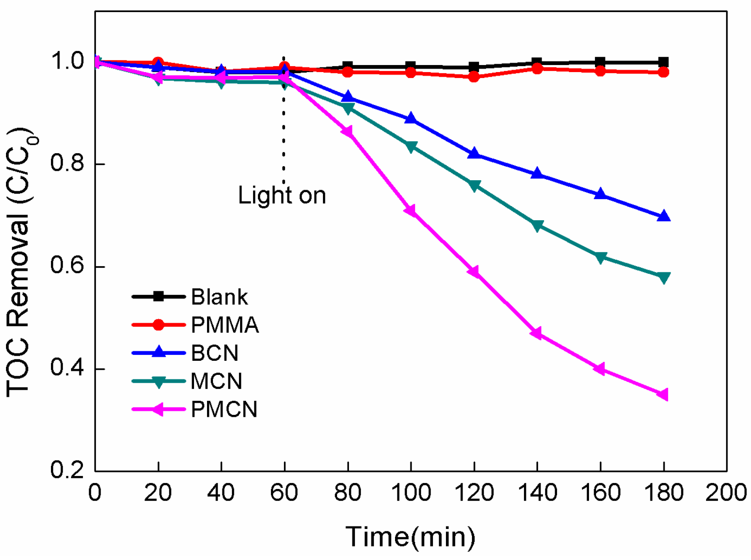

Figure S5. Photocatalytic activity of the as-prepared materials using phenol as the target pollutant

In the current study, the photocatalytic activity of PMCN was also evaluated by the colourless phenol, and found that its photodegradation efficiency under the visible light was much higher than those with MCN and BCN, indicating that the synthesized PMCN was an excellent visible-light photocatalyst.

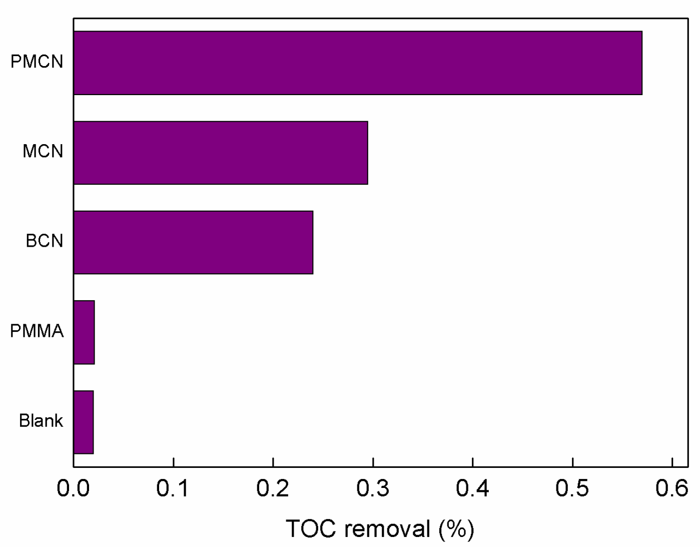

Figure S6. TOC removal of RhB by as-prepared photocatalysts under the irradiation of visible light

The removal efficiency of TOC was evaluated to deeper illuminate the degradation capabilities of our photocatalyst. Among all the samples, PMCN presented the most efficient photodegradation of RhB with a TOC removal efficiency of 57.0%, followed by MCN (29.5%) and BCN (24.0%). This experiment also showed that RhB failed to decompose without the photocatalysts, revealing PMCN is able to mineralize RhB during the decolourization process.

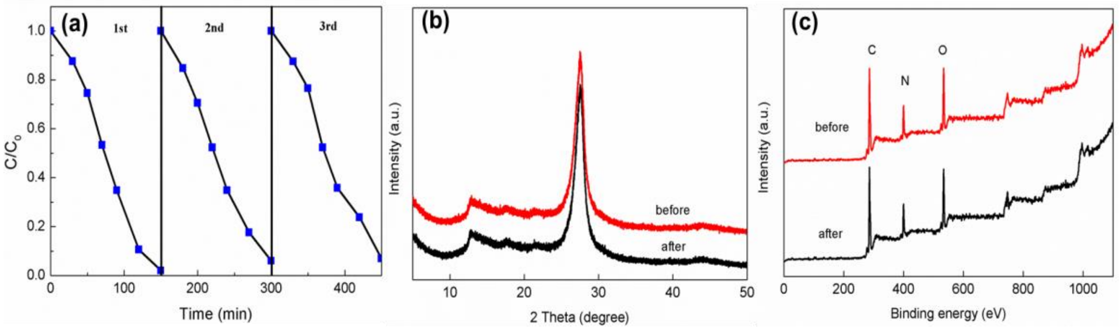

Figure S7. (a) Cycling runs during photocatalytic degradation of RhB in the presence of PMCN under visible light; Structural and compositional changes of PMCN before and after cycling tests (b) XRD patterns and (c) XPS surveys

The photocatalytic capability of recycled PMCN was evaluated to determine its photocatalytic stability. When the PMCN photocatalyst was used three times, its ability on RhB decolourization can maintain a relative high level. Moreover, both XRD and XPS spectra have revealed that there is neither structural nor compositional change occurring after the cycling tests. Therefore it can be confirmed from the results that MCN modified with PMMA exhibited good photocatalytic stability under visible light and the photocatalytic process has caused no significant changes in the structure or composition of the composite.

Reference

1. Shiraishi, Y. et al. Sunlight-driven hydrogen peroxide production from water and molecular oxygen by metal-free photocatalysts. *Angew. Chem. Int. Edit.* **53,** 13454-13459 (2014).
